# Bridging computational power and environmental challenges: a perspective on neural network predictive models for environmental engineering

**DOI:** 10.3389/frai.2025.1708369

**Published:** 2026-01-05

**Authors:** Jussen Facuy, Diego Arcos-Jacome

**Affiliations:** 1 Ingeniería Ambiental, Facultad de Ciencias Agrarias, Universidad Agraria del Ecuador, Guayaquil, Guayas, Ecuador; 2 Instituto de Investigación en Informática III-LIDI, Facultad de Informática, Universidad Nacional de la Plata, La Plata, Buenos Aires, Argentina

**Keywords:** artificial intelligence, mathematical models, environmental engineering, prediction, environmental management

## Abstract

The escalating frequency and severity of extreme environmental events underscores the critical need for a paradigm shift from reactive to proactive management strategies. This perspective article argues that artificial neural networks (ANNs) represent a transformative tool for environmental forecasting, capable of capturing the non-linear, high-dimensional dynamics that define complex Earth systems. While ANNs demonstrate superior predictive performance across domains such as hydrology, air quality, and ecology, their integration into decision-making workflows remains hindered by challenges related to data quality, model interpretability, and a lack of interdisciplinary collaboration. We synthesize current advancements, highlighting the pivotal role of physics-informed neural networks (PINNs) and explainable AI (XAI) in bridging the gap between data-driven insights and physical plausibility. Finally, we propose a concrete interdisciplinary roadmap, encompassing curated benchmarks, hybrid modeling, educational initiatives, and institutional co-design, to translate computational potential into trustworthy, actionable tools for building environmental resilience.

## 1 Introduction

The increasing frequency and severity of extreme environmental events, ranging from catastrophic floods to widespread wildfires, underscore the limitations of reactive management and the urgent need for reliable forecasting tools (IPCC, 2023). Predictive modeling has therefore become a cornerstone of proactive mitigation and adaptation strategies (Dikmen, 2025). However, the inherent complexity of environmental systems, characterized by nonlinear dynamics, high dimensionality, and intricate feedback loops, continues to challenge traditional modeling approaches (Eid, 2025). These challenges have direct implications for water security, agricultural productivity, public health, and the design of early warning systems fundamental to societal resilience (Farhangmehr, 2025).

Techniques, including ARIMA and multiple linear regression, as well as process-based mechanistic models, have long contributed to understanding hydrologic and atmospheric processes (He, 2023; Razavi, 2022). Yet these models often struggle to capture the full range of nonlinearities and multiscale interactions present in large and noisy environmental datasets (Molkov et al., 2022). Despite recent advances, a gap persists in fully leveraging modern computational capabilities to overcome these limitations. Deep learning applications, particularly neural networks, remain insufficiently integrated with domain knowledge, and issues of data quality, workflow standardization, and model interpretability continue to hinder trust and operational adoption (Janiesch et al., 2021; Xu, 2024).

This perspective argues that artificial neural networks (ANNs) offer a promising path toward next-generation environmental forecasting when coupled with robust data practices and interpretability frameworks. Rather than replacing domain expertise, ANNs should be guided by it to generate physically plausible, transparent, and operationally useful models. The article establishes the theoretical rationale for ANN-based modeling, highlights key application areas, discusses persistent challenges, and outlines an interdisciplinary roadmap for integrating neural networks into environmental engineering practice.

## 2 Theoretical rationale: why neural networks for environmental forecasting?

Artificial neural networks (ANNs) provide a flexible computational framework capable of approximating highly nonlinear and high-dimensional relationships that characterize environmental systems (Karniadakis et al., 2021; Razavi, 2021). Unlike conventional statistical or mechanistic models, which rely on predefined functional structures, ANNs learn representations directly from data, enabling them to capture complex spatiotemporal interactions and system behaviors that are difficult to specify analytically.

Recurrent architectures such as Long Short-Term Memory (LSTM) networks and gated units are particularly effective at modeling temporal dependencies, while convolutional structures extract spatial features relevant in hydrology, atmospheric science, and remote sensing (Sit et al., 2020; Zhang, 2021). Owing to these capabilities, ANNs serve not merely as statistical tools but as universal approximators capable of mapping intricate input–output relationships in large environmental datasets. When applied appropriately, especially in contexts with abundant data and strong nonlinear dynamics, ANNs can enable more accurate and adaptive environmental forecasting than linear or semi-empirical approaches.

## 3 Frontiers of application: key domains in environmental science

The application of neural networks has catalyzed a revolution across nearly every sub-discipline of environmental science, enabling predictions at unprecedented resolutions and accuracies (Boukabara, 2021; Zhong, 2021). In hydrology, LSTM networks have become a benchmark for rainfall-runoff modeling, often outperforming established conceptual models (Sit et al., 2020). Wajid et al. (2024) state in their research that floods are among the most devastating natural disasters and therefore propose an IoT based prediction model that analyzes seven factors and implements neural networks. They tested additional algorithms Logistic Regression and Decision Tree to compare their efficiency, obtaining accuracies of LR (89.6%), Random Forest (87.9%), and ANN (94.2%), demonstrating that ANNs outperform the other algorithms in terms of precision.

**Figure 1:**
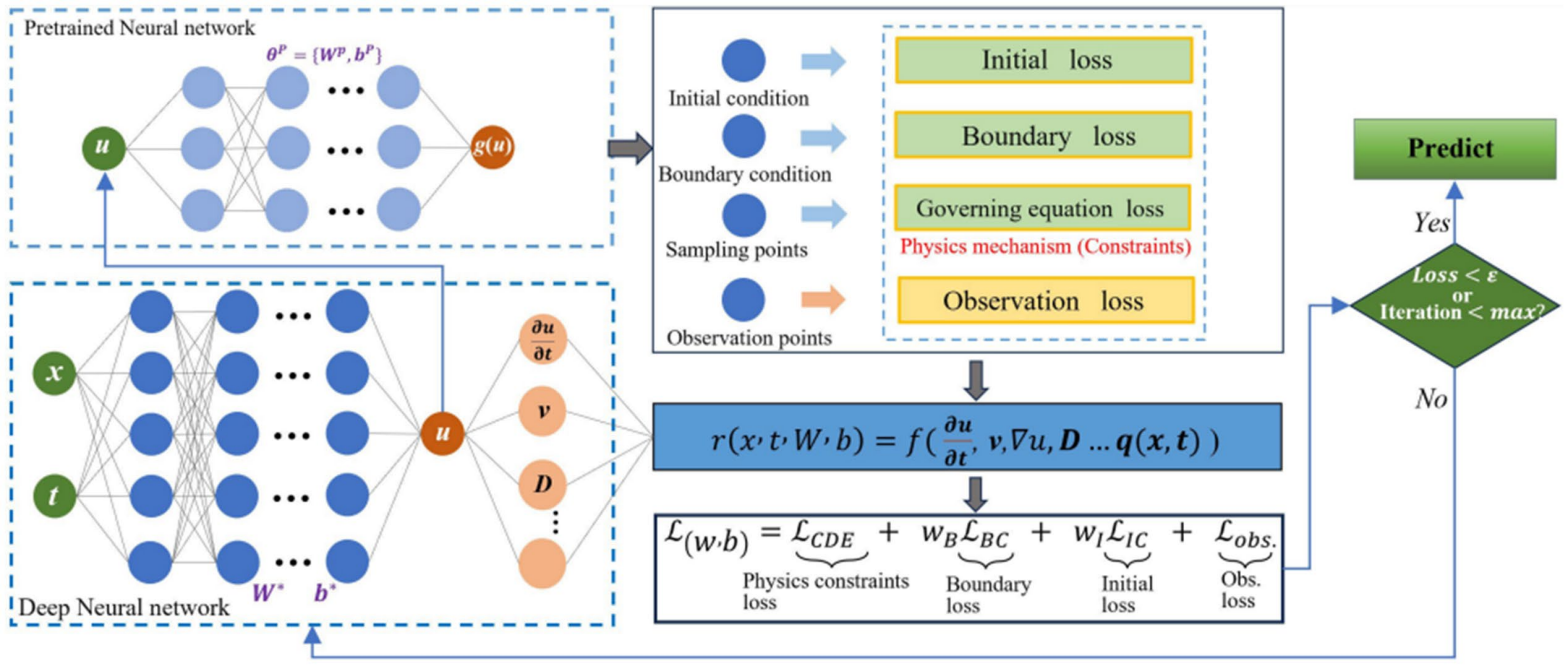
Physics-informed neural network (PINN). The network minimizes a global loss L that penalizes deviations from data ( $L_{\mathrm{obs}}$ ) and physical constraints, including the governing equation ( $L_{\mathrm{CDE}}$ ), boundary ( $L_{\mathrm{BC}}$ ), and initial conditions ( $L_{\mathrm{IC}}$ ). Adapted from Jia (2025).

Likewise, in the following Figure 1, (Jia, 2025) presents the schematic representation of the physics-informed neural network model for hydrodynamic evolution problems, demonstrating that HC-PDNet achieved the lowest errors, greater stability, and an improvement of up to 79.32% in accuracy compared to FNO, becoming the most efficient and robust model for predicting hydrodynamic diffusion fields.

For air quality forecasting, hybrid models combining CNNs for spatial feature extraction with LSTMs for temporal dynamics are setting new standards in predicting PM2.5 and O₃ concentrations (Zhang, 2021). Kamsing et al. (2025) state that the prediction of atmospheric pollutant concentrations (CO, NO_2_, PM2.5, and PM10) at Suvarnabhumi Airport can be accomplished using artificial neural networks. They proposed a model based on aircraft trajectories obtained from ADS-B data, analyzing eight input factors. Tests were conducted on four pollutants to evaluate the model’s efficiency, yielding R^2^ values of 0.4946 for CO, 0.3339 for NO_2_, 0.4762 for PM2.5, and 0.5594 for PM10, concluding that the model is acceptable for environmental management and route planning applications.

Beyond geophysics, in ecology, CNNs automate the analysis of camera trap and acoustic recordings, enabling large-scale biodiversity monitoring and species identification (Murray, 2022). Odilov et al. (2024)developed an IoT-based water-quality monitoring system to assess aquatic ecosystem health and preserve biodiversity, combined with a generalized regression neural network (G-RNN) to estimate water parameters that cannot be measured by sensors; they achieved high accuracy for nitrates and phosphates, with *R*^2^ values between 0.79 and 0.89 and errors below 0.3 mg/L, results the authors regarded as adequate and satisfactory.

The common thread across these diverse applications is the ability of ANNs to fuse heterogeneous data streams into a unified predictive framework, pushing the boundaries from isolated models to integrated digital twins of environmental systems (Hammoumi, 2025). The versatility of ANNs extends to urban waste management, where, for instance, Facuy et al. (2025) successfully applied an ambient intelligence system powered by neural networks to predict electronic waste generation, demonstrating its practical utility for logistical planning in smart cities. Future efforts must focus on developing transferable, pre-trained models that can be adapted to different regions, democratizing access to advanced predictive capabilities for global environmental challenges (Xiong, 2022). The qualitative results of these studies strengthen the credibility of the ANN approach and demonstrate its competitiveness compared with other predictive methods.

## 4 The data dilemma: challenges in quality, quantity, and Preprocessing

The performance of any neural network is fundamentally constrained by the quality, quantity, and structure of its training data, presenting a significant barrier in environmental contexts (Liu, 2022; Sambasivan et al., 2021). Environmental data is notoriously noisy, plagued by missing values from sensor failures, and often skewed by indirect measurement techniques (Sambasivan et al., 2021). While ANNs require large volumes of data for training, many critical environmental phenomena are rare events (e.g., extreme floods), leading to imbalanced datasets that bias models toward predicting the median state (Liu, 2022).

Furthermore, a lack of standardized data pre-processing protocols can lead to data leakage and overly optimistic performance estimates, undermining the reliability of published models (Sambasivan et al., 2021). This underscores that data curation is not a preliminary step but a central, intellectually demanding part of the modeling workflow, where the adage “garbage in, garbage out” is acutely relevant. Studies such as that of Wajid et al. (2024) show that artificial neural networks (ANN) achieve 94.2% accuracy in flood-prediction tasks, clearly outperforming traditional algorithms such as logistic regression (89.6%) and decision trees (87.9%).

We advocate for the development and adoption of rigorous benchmarking datasets and pre-processing pipelines specific to environmental data to ensure reproducibility and foster trust in model outcomes for critical environmental decision-making.

## 5 Beyond the black box: the imperative of interpretability and explainability (XAI)

The perceived “black box” nature of deep learning models is a major impediment to their adoption for high-stakes environmental decision-making and policy formulation (Xu, 2024). While ANNs provide accurate predictions, their complex inner workings often obscure how and why a specific prediction was made, eroding trust among stakeholders and regulators (Xu, 2024; Janiesch et al., 2021). In response, the field of Explainable AI (XAI) has developed techniques such as SHAP (SHapley Additive exPlanations) and LIME (Local Interpretable Model-agnostic Explanations) to attribute predictions to input features (Bao, 2025).

Emerging research shows that integrating physical principles directly into ANN architectures (e.g., physics-informed neural networks) not only improves extrapolation but also makes models more interpretable by aligning them with known laws of nature (Karniadakis et al., 2021; Jia, 2025). Interpretability should not be an afterthought but a core design objective, as an interpretable model provides not just a prediction but also scientific insight, potentially revealing previously unknown relationships within the environmental system. We strongly recommend that the evaluation criteria for environmental ML models be expanded beyond predictive accuracy to include metrics of interpretability and physical consistency to facilitate their adoption in policy and management contexts.

## 6 An interdisciplinary roadmap: integrating computation and domain expertise

The literature consistently emphasizes that realizing the full potential of neural networks in environmental science requires a genuinely interdisciplinary model integrating computational methods with domain-specific knowledge (Karniadakis et al., 2021; Boukabara, 2021). Evidence shows that the most effective ANN applications arise when environmental scientists frame and validate the problem, while data scientists develop and optimize the models, underscoring the complementary nature of both roles (Boukabara, 2021). This convergence has prompted educational programs to formalize “environmental data science” as an emerging hybrid discipline that cultivates expertise across these domains (McGovern et al., 2019).

Moreover, integrative frameworks such as theory-guided data science (TGDS) institutionalize this collaboration by embedding physical principles into neural network design, thereby enhancing both statistical robustness and physical plausibility (Karniadakis et al., 2021; Jia, 2025). The literature suggests that such synergy is essential: domain insight mitigates the risk of producing computationally sophisticated yet scientifically invalid models, while advanced ML techniques enable the detection of complex environmental patterns not accessible through traditional approaches. In line with these findings, several authors advocate for the development of interdisciplinary research clusters and curricula that dismantle disciplinary silos and support collaborative solution-building for the environmental problems highlighted in recent assessments (IPCC, 2023; Bennett et al., 2021).

## 7 Discussion

The growing severity of climate-driven extremes highlighted by recent IPCC findings (IPCC, 2023), has catalyzed a shift in the literature toward rethinking environmental forecasting as a proactive, data-centric enterprise. Across studies, artificial neural networks (ANNs) consistently emerge as a foundational technology enabling this transition, though our synthesis shows that their transformative potential depends less on computational capacity and more on resolving interdisciplinary challenges tied to data quality, interpretability, and the integration of physical principles into data-driven workflows (Liu, 2022; Wang, 2023).

The evidence consolidates three core dimensions of this integration. First, multiple investigations confirm the superior ability of ANN architectures especially LSTMs and CNNs to model the nonlinear, high-dimensional behavior of hydrological systems (Sit et al., 2020; Feng, 2023) and atmospheric processes (Zhang, 2021), reinforcing early arguments that deep learning would redefine Earth system science (Boukabara, 2021). Second, our comparative reading indicates that the primary limitation is no longer model design but the “data bottleneck”: a persistent deficit in standardized, high-quality environmental datasets, particularly for forecasting rare but high-impact extremes (Liu, 2022; Xiong, 2022). The absence of universal preprocessing protocols further undermines reproducibility and equitable model benchmarking, a problem widely noted across the field (Sambasivan et al., 2021).

Third, and emerging as the most decisive trend, the field is shifting from generic black-box modeling to frameworks that foreground interpretability and physical consistency. Recent critiques underscore that in high-stakes environmental contexts, explainability is essential for institutional trust and operational uptake (Xu, 2024). This demand is driving the adoption of physics-informed neural networks (PINNs) and related advances (Karniadakis et al., 2021), as well as the broader Theory-Guided Data Science (TGDS) paradigm (Jia, 2025), which collectively embed physical knowledge into ANN training to ensure that predictions remain both statistically robust and physically meaningful.

Recent literature suggests that advances in neural network–based environmental forecasting hinge on both technological refinement and sustained interdisciplinary collaboration. Technologically, although ANNs function as powerful universal approximators, their complexity necessitates deliberate architectural choices to prevent non-physical outputs. Within this context, research on transfer learning has emerged as a promising pathway for mitigating data scarcity in under-monitored regions, enabling models trained on data-rich basins to be effectively adapted elsewhere and thereby broadening access to high-quality predictive tools (Xiong, 2022). Yet, the efficacy of such approaches is shown to depend fundamentally on iterative cooperation between data scientists and domain experts.

Contrary to critiques that interdisciplinary processes introduce inefficiencies, our synthesis aligns with arguments that this negotiated co-design is indispensable: without it, data scientists risk optimizing solutions to mis-specified problems, while domain experts may disregard otherwise powerful tools they cannot adequately interpret. The Theory-Guided Data Science (TGDS) framework formalizes this symbiosis by providing conceptual and methodological scaffolding for integrating physical knowledge with data-driven modeling (Jia, 2025). Parallel insights from socio-technical systems research further reinforce that sustainability-oriented tools achieve greater legitimacy and equity when developed *with*, rather than merely *for*, their stakeholders (Bennett et al., 2021).

At the same time, a clear delineation of scope remains essential. While this perspective highlights the unique advantages of neural networks for high-dimensional spatiotemporal problems, other machine learning families especially tree-based ensemble methods such as XGBoost continue to perform at state-of-the-art levels for many tabular-data applications in environmental science (Grinsztajn et al., 2022; Janiesch et al., 2021). Accordingly, the conclusions drawn here apply specifically to domains where the strengths of neural networks are most pertinent. Because this contribution synthesizes existing literature rather than presenting new empirical evidence, it does not claim ANN superiority across all contexts but instead positions them as particularly powerful for a specific subset of forecasting challenges. Nonetheless, the persistent absence of standardized, community-endorsed benchmarks for datasets and evaluation protocols remains a critical barrier to producing field-wide, generalizable insights (Sambasivan et al., 2021).

To translate this perspective into tangible progress, we propose a concerted agenda for future research and practice:

1. Creation of curated benchmark datasets: A community-wide initiative to develop and maintain public datasets for flagship challenges (e.g., predicting compound extremes, pollutant transport) with rigorous pre-processing standards and evaluation metrics. This is a prerequisite for reproducibility and fair model comparison, as pioneered in related fields (Sambasivan et al., 2021).
2. Advancement of hybrid, transferable, and interpretable models: Prioritizing research that integrates physical laws (via PINNs, TGDS), enhances explainability (XAI), and leverages transfer learning to address data inequality. Success must be measured by performance on benchmarks, physical consistency, operational utility, and the ability to provide scientific insight (Karniadakis et al., 2021; Xiong, 2022).
3. Cultivation of interdisciplinary capacity: The active promotion of “environmental data science” as a discipline through dedicated academic programs and continuous learning platforms to train a generation of professionals fluent in both domain knowledge and computational methods (Zhong, 2021).
4. Institutionalization of Co-Design: Funding agencies and research institutions must create mechanisms and incentives that mandate the formation of deeply integrated teams from project inception, ensuring tools are co-designed with end-users to guarantee their practical relevance and adoption (Bennett et al., 2021).

Beyond their technical performance, the deployment of AI-based environmental models carries important ethical and policy implications that must be considered in decision-making contexts. Environmental datasets often reflect spatial, socioeconomic, and infrastructural inequalities, which may introduce bias in model predictions and disproportionately affect vulnerable communities. Furthermore, when ANN-based forecasts are used to guide risk-management actions, the lack of transparency and interpretability may limit accountability and reduce stakeholder trust. These concerns underscore the need for governance frameworks promoting explainability, equitable data practices, and responsible use of automated predictions in climate resilience and environmental planning.

In conclusion, bridging the chasm between computational potential and environmental problem-solving is a quintessential socio-technical challenge. It requires building a shared language and purpose across the entire pipeline from sensor deployment and data curation to model development and decision-making. By championing a culture of collaboration centered on robust data, transparent models, and shared goals, neural networks can mature from powerful academic exercises into indispensable, trustworthy partners in the urgent global effort to build resilience and ensure sustainability.

## Data Availability

The original contributions presented in the study are included in the article/supplementary material, further inquiries can be directed to the corresponding author.

## References

[ref1] Bao X. (2025). Carbon emission prediction and characteristic analysis of railway ballastless track using AutoGluon-XAI. J. Saf. Environ. 25, 2431–2440. doi: 10.13637/j.issn.1009-6094.2024.1709

[ref2] Bennett N. J. Blythe J. White C. S. Campero C. (2021). Blue growth and blue justice: ten risks and solutions for the ocean economy. Mar. Policy 125:104387. doi: 10.1016/j.marpol.2020.104387

[ref3] Boukabara S. A. (2021). Outlook for exploiting artificial intelligence in the earth and environmental sciences. Bull. Am. Meteorol. Soc. 102, E1016–E1032. doi: 10.1175/BAMS-D-20-0031.1

[ref4] Dikmen F. (2025). AI-driven wastewater management through comparative analysis of feature selection techniques and predictive models. Sci. Rep. 15:25347. doi: 10.1038/s41598-025-07124-0, 40659650 PMC12259835

[ref5] Eid A. (2025). Machine learning-based analysis of workers’ exposure and detection to volatile organic compounds (VOC). Int. J. Environ. Sci. Technol. 22, 12385–12401. doi: 10.1007/s13762-025-06355-y

[ref6] Facuy J. Aguirre-Munizaga M. Pasini A. Estévez E. Moran-Castro C. (2025). “Validation of an ambient intelligence system applied to the prediction of electronic waste in smart cities” in Technologies and Innovation. CITI 2025. Communications in Computer and Information Science. ed. Valencia-Garcia R., vol. 2776 (Cham: Springer), 259–272.

[ref7] Farhangmehr V. (2025). A spatiotemporal CNN-LSTM deep learning model for predicting soil temperature in diverse large-scale regional climates. Sci. Total Environ. 968:8901. doi: 10.1016/j.scitotenv.2025.178901, 39987832

[ref8] Feng Z. (2023). Road noise prediction model with CEEMD-GRU combination. J. Saf. Environ. 23, 2128–2136. doi: 10.13637/j.issn.1009-6094.2022.0715

[ref9] Grinsztajn L. Oyallon E. Varoquaux G. (2022). Why do tree-based models still outperform deep learning on tabular data? Adv. Neural Inf. Process. Syst. 35, 507–520. doi: 10.48550/arXiv.2207.08815

[ref10] Hammoumi A. (2025). Digital twin generation for adsorption in porous materials using stochastic MorphoDeep. Commun. Mater. 6:179. doi: 10.1038/s43246-025-00906-z

[ref11] He C. (2023). A hybrid model based on multi-LSTM and ARIMA for time series forcasting. 2023 8th International Conference on Intelligent Computing and Signal Processing (ICSP). Xi'an, China: IEEE. 612–616.

[ref12] IPCC (2023). “Index” in Climate change 2022 – Impacts, adaptation and vulnerability (Cambridge: Cambridge University Press), 3005–3056.

[ref13] Janiesch C. Zschech P. Heinrich K. (2021). Machine learning and deep learning. Electron. Mark. 31, 685–695. doi: 10.1007/s12525-021-00475-2

[ref14] Jia L. (2025). A physics-coupled deep learning framework for hydrodynamic diffusion modeling in watershed systems: integrating spatiotemporal networks and environmental constraints. IEEE Access 13, 34985–35003. doi: 10.1109/ACCESS.2025.3542173

[ref15] Kamsing P. Cao C. Boonpook W. Boonprong S. Xu M. Boonsrimuang P. (2025). Artificial neural network for air pollutant concentration predictions based on aircraft trajectories over Suvarnabhumi international airport. Atmos. 16:366. doi: 10.3390/ATMOS16040366

[ref16] Karniadakis G. E. Kevrekidis I. G. Lu L. Perdikaris P. Wang S. Yang L. (2021). Physics-informed machine learning. Nat. Rev. Phys. 3, 422–440. doi: 10.1038/s42254-021-00314-5

[ref17] Liu X. (2022). Data-driven machine learning in environmental pollution: gains and problems. Environ. Sci. Technol. 56, 2124–2133. doi: 10.1021/acs.est.1c06157, 35084840

[ref18] McGovern A. Lagerquist R. John Gagne D. Jergensen G. E. Elmore K. L. Homeyer C. R. . (2019). Making the black box more transparent: understanding the physical implications of machine learning. Bull. Am. Meteorol. Soc. 100, 2175–2199. doi: 10.1175/BAMS-D-18-0195.1

[ref19] Molkov A. Fedorov S. Pelevin V. (2022). Toward atmospheric correction algorithms for Sentinel-3/OLCI images of productive waters. Remote Sens 14:3663. doi: 10.3390/rs14153663

[ref20] Murray X. (2022). Rapid assessment of mine rehabilitation areas with airborne LiDAR and deep learning: bauxite strip mining in Queensland, Australia. Geocarto Int. 37, 11223–11252. doi: 10.1080/10106049.2022.2048902

[ref21] Odilov B. A. Madraimov A. Yusupov O. Y. Karimov N. R. Alimova R. Yakhshieva Z. Z. . (2024). Utilizing deep learning and the internet of things to monitor the health of aquatic ecosystems to conserve biodiversity. Nat. Eng. Sci. 9, 72–83. doi: 10.28978/NESCIENCES.1491795

[ref22] Razavi S. (2021). Deep learning, explained: fundamentals, explainability, and bridgeability to process-based modelling. Environ. Model. Softw. 144:5159. doi: 10.1016/j.envsoft.2021.105159

[ref23] Razavi S. (2022). Coevolution of machine learning and process-based modelling to revolutionize earth and environmental sciences: a perspective. Hydrol. Process. 36:4596. doi: 10.1002/hyp.14596

[ref24] Sambasivan N. Kapania S. Highfill H. Akrong D. Paritosh P. Aroyo L. M. (2021). “Everyone wants to do the model work, not the data work”: Data Cascades in High-Stakes AI. Proceedings of the 2021 CHI Conference on Human Factors in Computing Systems. New York, NY, USA: ACM, 1–15.

[ref25] Sit M. Demiray B. Z. Xiang Z. Ewing G. J. Sermet Y. Demir I. (2020). A comprehensive review of deep learning applications in hydrology and water resources. Water Sci. Technol. 82, 2635–2670. doi: 10.2166/wst.2020.369, 33341760

[ref26] Wajid M. Abid M. K. Raza A. A. Haroon M. Mudasar A. Q. (2024). Flood prediction system using IOT & artificial neural network. VFAST Transactions on Software Engineering 12, 210–224. doi: 10.21015/VTSE.V12I1.1603

[ref27] Wang H. (2023). Spatio-temporal fusion of meteorological factors for multi-site PM2.5 prediction: a deep learning and time-variant graph approach. Environ. Res. 239:7286. doi: 10.1016/j.envres.2023.117286 37797668

[ref28] Xiong R. (2022). Predicting dynamic riverine nitrogen export in unmonitored watersheds: leveraging insights of AI from data-rich regions. Environ. Sci. Technol. 56, 10530–10542. doi: 10.1021/acs.est.2c02232, 35772808

[ref29] Xu H. (2024). Trusted artificial intelligence for environmental assessments: an explainable high-precision model with multi-source big data. Environ. Sci. Ecotechnol. 22:479. doi: 10.1016/j.ese.2024.100479, 39286480 PMC11402945

[ref30] Zhang B. (2021). A novel encoder-decoder model based on read-first LSTM for air pollutant prediction. Sci. Total Environ. 765:4507. doi: 10.1016/j.scitotenv.2020.144507, 33418334

[ref31] Zhong S. (2021). Machine learning: new ideas and tools in environmental science and engineering. Environ. Sci. Technol. 55, 12741–12754. doi: 10.1021/acs.est.1c01339, 34403250

